# Yoga Posture Recognition and Quantitative Evaluation with Wearable Sensors Based on Two-Stage Classifier and Prior Bayesian Network

**DOI:** 10.3390/s19235129

**Published:** 2019-11-23

**Authors:** Ze Wu, Jiwen Zhang, Ken Chen, Chenglong Fu

**Affiliations:** 1 Department of Mechanical Engineering, Tsinghua University, Beijing 100084, China; wuz17@mails.tsinghua.edu.cn (Z.W.); jwzhang@mail.tsinghua.edu.cn (J.Z.); kenchen@tsinghua.edu.cn (K.C.); 2 Department of Mechanical and Energy Engineering, Southern University of Science and Technology, Shenzhen 518055, China

**Keywords:** yoga posture recognition and evaluation, inertial measurement unit, BP-ANN and FCM, Bayesian network

## Abstract

Currently, with the satisfaction of people’s material life, sports, like yoga and tai chi, have become essential activities in people’s daily life. For most yoga amateurs, they could only learn yoga by self-study, like mechanically imitating from yoga video. They could not know whether they performed standardly without feedback and guidance. In this paper, we proposed a full-body posture modeling and quantitative evaluation method to recognize and evaluate yoga postures to provide guidance to the learner. Back propagation artificial neural network (BP-ANN) was adopted as the first classifier to divide yoga postures into different categories, and fuzzy C-means (FCM) was utilized as the second classifier to classify the postures in a category. The posture data on each body part was regarded as a multidimensional Gaussian variable to build a Bayesian network. The conditional probability of the Gaussian variable corresponding to each body part relative to the Gaussian variable corresponding to the connected body part was used as criterion to quantitatively evaluate the standard degree of body parts. The angular differences between nonstandard parts and the standard model could be calculated to provide guidance with an easily-accepted language, such as “lift up your left arm”, “straighten your right forearm”. To evaluate our method, a wearable device with 11 inertial measurement units (IMUs) fixed onto the body was designed to measure yoga posture data with quaternion format, and the posture database with a total of 211,643 data frames and 1831 posture instances was collected from 11 subjects. Both the posture recognition test and evaluation test were conducted. In the recognition test, 30% data was randomly picked from the database to train BP-ANN and FCM classifiers, and the recognition accuracy of the remaining 70% data was 95.39%, which is highly competitive with previous posture recognition approaches. In the evaluation test, 30% data were picked randomly from subject three, subject four, and subject six, to train the Bayesian network. The probabilities of nonstandard parts were almost all smaller than 0.3, while the probabilities of standard parts were almost all greater than 0.5, and thus the nonstandard parts of body posture could be effectively separated and picked for guidance. We also tested separately the trainers’ yoga posture performance in the condition of without and with guidance provided by our proposed method. The results showed that with guidance, the joint angle errors significantly decreased.

## 1. Introduction

With the satisfaction of people’s material life, people are pursuing spiritual level and focusing on body health, and as a result, sports, like yoga and tai chi, have become essential activities in people’s daily life. Yoga is one of the most popular sports in the world, while the professional yoga instructors are only a very few. For most yoga amateurs, they could only learn yoga by self-study, like mechanically imitating from yoga video. In this way, it is difficult for the learner to observe the posture details of their whole body, since most postures require the learner to look at in a certain direction, and some postures even need the learner to lie on their stomach or back. As a result, they can not know whether they performed standardly or not. Consequently, it would cause low efficiency of learning. Hence, yoga posture recognition and evaluation are significant for guidance to self-study.

Yoga postures involve all parts of the body, which would increase the difficulty of posture acquisition and representation. Additionally, due to the large differences in the physical qualities of different trainers, such as flexibility of the body and ability to stretch the joints, the body shapes of different trainers performing the same yoga posture are also quite different. These differences greatly increase the difficulty of posture recognition. Previous human–robot interaction (HRI) research was mainly focused on posture recognition, while the posture evaluation was rarely studied. However, posture evaluation is significant in human sports as well. Posture evaluation could be used to find out about nonstandard parts of the body, which include misaligned limbs with respect to the standard posture model, such as the legs not being raised to the right height, the body not being stretched enough, or the joints not being maintained with the right angle.

Meanwhile, posture evaluation can be a challenging problem, since it is complex to quantitatively describe the standard degree of body parts in a posture. In addition, it is also an issue to translate the guidance information to daily language.

The information acquisition of full-body posture is the basis of posture recognition and evaluation. Many devices have been developed to acquire posture information with various sensors. The most common sensors are image sensor [1,2,3,4], electromyogram sensor [5,6,7,8], wearable receiving tags [9], and inertial measurement units (IMUs) [10,11,12,13,14]. In general, with image sensors, people need not wear an extra device, but the image background should be simple and the body posture cannot overlap to produce image occlusion. The electromyogram (EMG) signal is easily interfered by noise, especially the power frequency, and the valid signal intensity is generally low. These factors cause the low signal–noise ratio (SNR). The IMU device is small and precise. Since yoga postures involve all parts of the body and most of them have body folding, IMU sensor is more suitable for data acquisition of yoga posture.

A lot of human posture modeling researches have been developed in recent years. Hidden Markov model (HMM), support vector machine (SVM), decision tree (DT), and template map are the most widely used methods. Yamato et al. [15] and Oh et al. [16] adopted HMM to model tennis batting postures and upper-body posture, respectively. Good average recognition accuracy was achieved. However, they only modeled a very few postures. Mo et al. [17], Zhao et al. [18], and Jae-Wan et al. [19] applied SVM to recognize human daily behaviors with body image sequences, and good recognition results have been achieved. Limited by the image sensors, only a few postures were modeled. Kang et al. [20] and Saha et al. [21] applied DT to classify human postures by representative features, such as joint angle and joint distance extracted by body skeleton model. Although the computing cost was not high, the recognition accuracy was relatively low. Luo et al. [10] configured 16 IMUs on human body to measure 15 joint angles. Chen et al. [22] separately placed two Kinetics in front and to the side of the trainer, which were used to acquire the binarization of posture images and extract human body contours. Postures were recognized by calculating the similarity between the trainer’s posture and the standard posture model. These two recognition methods did not select representative features nor utilize the combination of features, and it is not surprising that these methods did not achieve good recognition results.

A few researches have also been developed about posture evaluation. Wu et al. [23] proposed three criteria, namely joint angle, arm orientation and joint movement type, which could be used as the criterion to evaluate the forearm and upper arm. Hachaj et al. [24] proposed a posture description language (GDL) to redefine human postures and evaluate full body movements. These evaluation methods could pick the nonstandard body parts. However, they were hard to quantitatively evaluate the nonstandard body parts, and they have not yet been applied to full-body posture evaluation.

In order to model multiple full-body postures with high accuracy and fill the blank in the posture evaluation, we proposed a full-body posture modeling and quantitative evaluation method to recognize and evaluate yoga postures. A wearable device was designed with 11 IMUs fixed on body to measure human posture data with quaternion format. In the modeling stage, a two-stage classifier was adopted to model common full-body yoga postures. Back propagation artificial neural network (BP-ANN) was adopted as the first classifier to divide yoga postures into different categories, and fuzzy C-means (FCM) was utilized as the second classifier to classify the postures in a category. The quaternion data measured by IMU fixed on each body part was regarded as a multidimensional Gaussian variable to build Bayesian network. The conditional probability of Gaussian variable corresponding to each body part relative to Gaussian variable corresponding to the connected body part, was used as criterion to quantitatively evaluate the standard degree of various body parts compared with standard posture model. For example, we can evaluate the standard degree of forearm relative to upper arm, shank relative to thigh, upper arm relative breast, and thigh relative to waist. Guidance was provided to the learner with an easily-accepted language after evaluation, including correcting orientation and extent. Consequently, the learners’ nonstandard body parts could be indicated and corrected.

The rest of this paper is organized as follows. We firstly introduce the wearable device and the modeled yoga postures in Section 2. Section 3 and Section 4 will introduce the proposed posture modeling and recognition method and evaluation method respectively in detail. Our experimental results and discussion will be presented in Section 5. Finally, the conclusions and future work are laid out in Section 6.

## 2. Yoga Posture Database Capture

### 2.1. System Introduction

**Figure 1:**
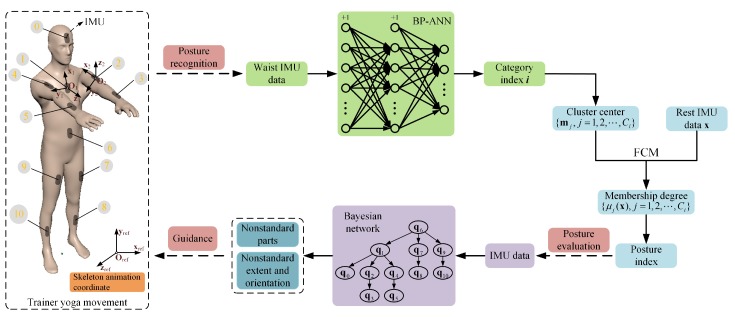
A general overview of yoga posture recognition and evaluation method. Eleven IMUs were fixed on the human body, and each IMU is marked with the index (0–10). $O_{1}-x_{1}y_{1}z_{1}$ is the local coordinate systems of IMU 0, 1, 6, 7, 8, 9, and 10, while $O_{2}-x_{2}y_{2}z_{2}$ is the local coordinate systems of IMU 2, 3, 4, and 5. $O_{\mathrm{ref}}-x_{\mathrm{ref}}y_{\mathrm{ref}}z_{\mathrm{ref}}$ is the coordinate system of skeleton animation. The yoga posture shown in the left is the calibration posture for mapping the trainer posture into the skeleton animation.

According to the advice of professional yoga instructors, most yoga postures are to stretch the neck, shoulders, waist, hip, arms, and legs, and the most commonly trained joints in yoga posture are elbow, hip, knee and shoulder. In order to ensure the measured posture data could be used to fully cover these parts, eleven IMUs are fixed on head, breast, left upper arm (LUArm), left forearm (LFArm), right upper arm (RUArm), right forearm (RFArm), waist, left thigh (LThigh), left shank (LShank), right thigh (RThigh), and right shank (RShank), as shown in Figure 1. These IMUs could richly express the state of corresponding body part. Since eleven IMUs are sufficient to provide data to model common yoga postures and calculate the most commonly trained joint angles, there is no need to add extra IMUs. The IMU data was used to extract features and recognize the full-body yoga postures by employing the pre-trained two-stage classifier, which contains BP-ANN and FCM. When a yoga posture was recognized, the pre-built prior Bayesian network was activated to calculate the standard degree of all body parts. And then the body parts whose standard degree was below a given threshold could be picked as nonstandard body parts. If there existed some body parts in the recognized posture been picked as nonstandard body parts, the deviated extent and orientation between nonstandard body parts and standard model were calculated as guidance to provide to learner with an easily-accepted language. Hence, the learner could be guided to perform properly.

### 2.2. Wearable Device

Our wearable device includes 11 IMUs (MTw Awinda (Xsens, Enschede, The Netherlands)). The IMU local coordinate systems are remapped to be consistent with the respective body part coordinate systems. Each IMU may transmit the orientation data, which could represent the orientations of IMU local coordinate systems in the global coordinate system, in the form of quaternion to a computer with an optional sampling frequency (40 Hz in this paper). The raw IMU quaternion data are expressed in $q_{0-\mathrm{global}}$, $q_{1-\mathrm{global}}$, ⋯, $q_{10-\mathrm{global}}$ according to the definition of quaternion rotation [25].

In order to display the trainer’s yoga posture intuitively, a virtual human skeleton animation is implemented to reproduce the trainer’s posture in real time. The posture calibration is necessary before the training begins. The calibration posture is shown in the left of Figure 1. When in calibration, the 11 IMU quaternion data will be recorded as $q_{\mathrm{cali},0-\mathrm{global}}$, $q_{\mathrm{cali},1-\mathrm{global}}$, ⋯, $q_{\mathrm{cali},10-\mathrm{global}}$. As shown in Figure 1, the local coordinate systems of IMUs fixed on head, breast, waist, thigh, and shank, are expressed in $O_{1}-x_{1}y_{1}z_{1}$, while the local coordinate systems of IMUs fixed on forearm and upper arm, are expressed in $O_{2}-x_{2}y_{2}z_{2}$. The local coordinate systems of $O_{1}-x_{1}y_{1}z_{1}$ and $O_{2}-x_{2}y_{2}z_{2}$ relative to the coordinate system of skeleton animation (expressed in $O_{\mathrm{ref}}-x_{\mathrm{ref}}y_{\mathrm{ref}}z_{\mathrm{ref}}$) were calculated in advance as $q_{\mathrm{init}1-\mathrm{interface}}=\left(0,\frac{\sqrt{2}}{2},\frac{\sqrt{2}}{2},0\right)$ and $q_{\mathrm{init}2-\mathrm{interface}}=\left(\frac{1}{2},-\frac{1}{2},-\frac{1}{2},-\frac{1}{2}\right)$, respectively.

Furthermore, the axis coordinate systems of IMUs fixed on body parts in the recognition process can be transformed into a coordinate system of skeleton animation with quaternion operation, which are expressed in $\left\{q_{i-\mathrm{interface}},\forall i=0,1,\cdots ,10\right\}$.

(1) $$q_{i-\mathrm{interface}}=q_{\mathrm{init}1-\mathrm{interface}}⊗q_{\mathrm{cali},i-\mathrm{global}}^{-1}⊗q_{i-\mathrm{global}},\;\;\forall i\in \left\{0,1,6,7,8,9,10\right\}$$

(2) $$q_{i-\mathrm{interface}}=q_{\mathrm{init}2-\mathrm{interface}}⊗q_{\mathrm{cali},i-\mathrm{global}}^{-1}⊗q_{i-\mathrm{global}},\;\;\forall i\in \left\{2,3,4,5\right\}$$

where $q_{i-\mathrm{global}}$ is the quaternion acquired by IMUs fixed on body parts in real time.

### 2.3. Posture and Subjects

**Figure 2:**
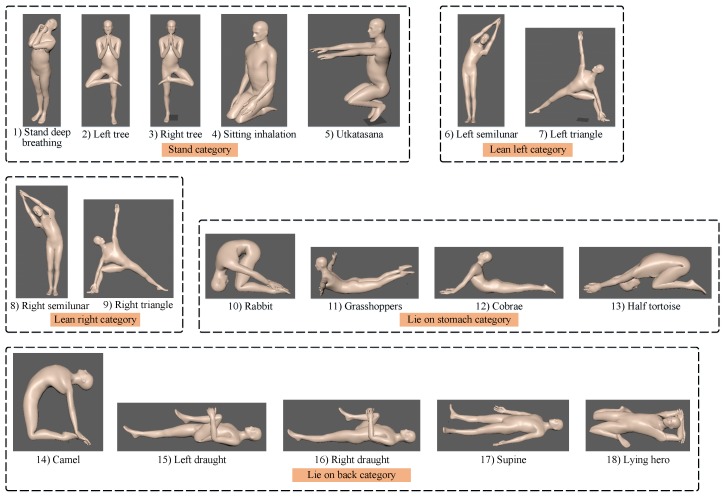
The illustration of 18 common yoga postures used in full-body posture recognition and evaluation.

In this study, eighteen common yoga postures were modeled as shown in the Figure 2. All postures were static. Based on the local waist IMU coordinate system relative to skeleton animation coordinate system, eighteen postures were divided into five categories in advance. Postures 1–5 belonged to the “stand” category. Postures 6 and 7 belonged to the “lean left” category. Postures 8 and 9 belonged to the “lean right” category. Postures 10–13 belonged to the “lie on stomach” category. Postures 14–18 belonged to the “lie on back” category.

The posture database was collected from 11 subjects (ten females and one male, aged 24–34). Among the 11 subjects, subject two, subject three, subject five, and subject six had learned yoga before, and the others had never learned yoga. Each subject was required to perform every yoga posture about 10 times with a random order and interval, and the posture labels were also recorded for the following recognition and evaluation. The database contained in total 211,643 data frames and 1831 posture instances.

## 3. Posture Modeling and Recognition

### 3.1. Posture Modeling

The body shapes of different trainers performing the same yoga posture were quite different, which could result in large intra-class difference and small interclass difference. Therefore, it was difficult to linearly distinguish yoga postures in the original feature space, and nonlinear mapping was necessary. BP-ANN was adopted to model yoga postures in this research. However, if only BP-ANN was utilized to model yoga postures with 11 IMU data, the modeling and recognition process was high in cost. As a result, we proposed a two-stage classifier and novel recognition method to decrease the computing cost with still-high recognition accuracy.

**Figure 3:**
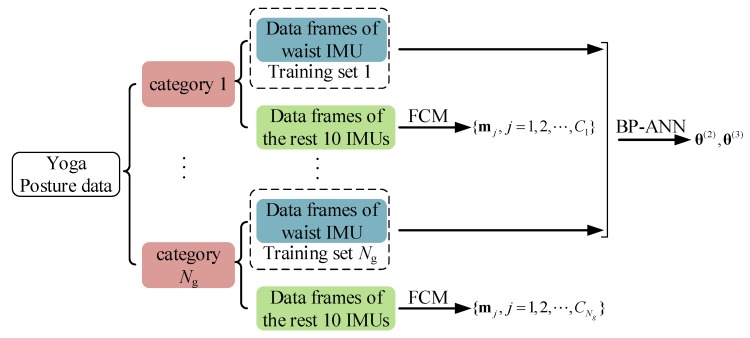
A general overview of the two-stage classifier. The data of waist IMU from all categories ($N_{g}=5$) were used to train the BP-ANN classifier, while the data of the rest ten IMUs from all postures in a specific category were used to train the corresponding FCM classifier.

As shown in Figure 3, the posture modeling method contained a two-stage classifier, which contained BP-ANN and FCM. Eighteen yoga postures were divided to different categories. The data of the waist IMU from all categories were used to train the BP-ANN classifier, while the data of the rest IMUs (all IMUs except the waist IMU) from all postures in a specific category were used to train the corresponding FCM classifier.

BP-ANN with three layers was designed to find the optimal feature combination in waist IMU data. The input layer had nine artificial neurons, the hidden layer had 15 artificial neurons, and the output layer has five artificial neurons, which corresponded to five posture categories. Bias unit was added both in the input layer and hidden layer. The commonly used activation function is sigmoid function that is defined as $\sigma \left(x\right)=\frac{1}{1+e^{-x}}$, which can map a real number into (0, 1). In this paper, the input of sigmoid function was the weighted sum of internal values of the artificial neurons in last layer $o^{\left(i-1\right)}$, and the output of the function was new internal states of the artificial neurons in current layer $o^{\left(i\right)}$.

(3) $$o_{j}^{\left(i\right)}=\sigma \left({\left(\theta_{j}^{\left(i\right)}\right)}^{T}O^{\left(i-1\right)}\right)=\frac{1}{1+e^{-{\left(\theta_{j}^{\left(i\right)}\right)}^{T}O^{\left(i-1\right)}}}$$

where $o_{j}^{\left(i\right)}$ is the jth artificial neuron’s value in the ith layer, $o^{\left(i-1\right)}$ is the artificial neuron’s value vector in the (i−1)th layer, $O^{\left(i-1\right)}={\left(1,o^{\left(i-1\right)}\right)}^{T}$ is the input of sigmoid function including bias unit, and $\theta_{j}^{\left(i\right)}$ is the weight vector from the (i−1)th layer to the jth artificial neuron in the ith layer.

As a result, the final output neuron’s values are decided by the values of artificial neurons $o^{\left(1\right)}$ in the input layer and the weights $\theta^{\left(2\right)}$, $\theta^{\left(3\right)}$ in the hidden layer and output layer.

The feature vector x in the input layer is nine components of the rotation matrix in the local waist IMU coordinate system relative to the coordinate system of the skeleton animation $q_{6-\mathrm{interface}}$.

(4) $$q_{6-\mathrm{interface}}⟹\left(\begin{array}{ccc} R_{11}^{\left(6\right)} & R_{12}^{\left(6\right)} & R_{13}^{\left(6\right)}\\ R_{21}^{\left(6\right)} & R_{22}^{\left(6\right)} & R_{23}^{\left(6\right)}\\ R_{31}^{\left(6\right)} & R_{32}^{\left(6\right)} & R_{33}^{\left(6\right)} \end{array}\right)$$

(5) $$x={\left\{R_{11}^{\left(6\right)},R_{12}^{\left(6\right)},R_{13}^{\left(6\right)},R_{21}^{\left(6\right)},R_{22}^{\left(6\right)},R_{23}^{\left(6\right)},R_{31}^{\left(6\right)},R_{32}^{\left(6\right)},R_{33}^{\left(6\right)}\right\}}^{T}$$

In the multi-classification case, the dependent variable y in the output layer was a $K_{1}$-dimensional one-hot vector. If the data frame was from the target posture, the corresponding component of y was set to 1 and others are set to 0.

Given the training set $\left\{\left(x^{\left(i\right)},y^{\left(i\right)}\right),i=1,2,\cdots ,m,y\in R^{K_{1}}\right\}$, which contained *m* samples, the BP-ANN process aimed to obtain the optimal weight parameters $\theta^{\left(2\right)}$, $\theta^{\left(3\right)}$ that minimized the cross-entropy cost function J(θ). In order to avoid over-fitting, it is common to introduce regularization term into the cost function. In this paper, L2 regularization was adopted, and thus the cost function with L2 regularization was defined as

(6) $$J\left(\theta \right)=-\frac{1}{m}\left[\sum_{i=1}^{m}\sum_{k=1}^{K_{1}}y_{k}^{\left(i\right)}\log\left({\left(o^{\left(3\right)}\right)}_{k}^{\left(i\right)}\right)+\left(1-y_{k}^{\left(i\right)}\right)\log\left(1-{\left(o^{\left(3\right)}\right)}_{k}^{\left(i\right)}\right)\right]+\frac{\lambda }{2m}\sum_{l=2}^{L}\sum_{i=1}^{s_{l}}\sum_{j=1}^{s_{l+1}}{\left(\theta_{i,j}^{\left(l\right)}\right)}^{2}$$

where ${\left(o^{\left(3\right)}\right)}_{k}^{\left(i\right)}$ is the kth artificial neuron’s value in the output layer with respect to the ith sample, and $K_{1}$ is the category number, which is set to 5 in this paper, *L* is the layer number which is set to 3 in this paper, $s_{l}$ is the artificial neuron number in the lth layer, and λ is regularization coefficient which reflects the reliability of trained weights approaching the optimal solution. Generally, the regularization term may not include the weights with respect to bias elements.

The training process was conducted by employing a back propagation (BP) algorithm, which began with a randomly initialized weight vector, and the optimal weights $\theta^{\left(2\right)},\theta^{\left(3\right)}$ could be obtained by repeated iteration up to convergence.

Due to the large intra-class difference and small interclass difference, it was not suitable to adopt the hard threshold, which may have caused poor recognition result. FCM was applied to the second classification instead. The basic idea of FCM was to make the similarity between samples classified into the same cluster become the largest, and make the similarity between samples classified to different clusters become the smallest.

In this study, the feature vector $x_{2}$ is the components of the rotation matrix in the rest local IMU coordinate systems relative to the local waist IMU coordinate system $\left\{q_{i-6},\forall i=0,1,\cdots ,10\;\mathrm{and}\;i\neq 6\right\}$, and therefore the feature vector is 90-dimensional vector.

(7) $$q_{i-6}=q_{6-\mathrm{interface}}^{-1}⊗q_{i-\mathrm{interface}}⟹\left(\begin{array}{ccc} R_{11}^{\left(i-6\right)} & R_{12}^{\left(i-6\right)} & R_{13}^{\left(i-6\right)}\\ R_{21}^{\left(i-6\right)} & R_{22}^{\left(i-6\right)} & R_{23}^{\left(i-6\right)}\\ R_{31}^{\left(i-6\right)} & R_{32}^{\left(i-6\right)} & R_{33}^{\left(i-6\right)} \end{array}\right)\;\forall i=0,1,\cdots ,10\;\mathrm{and}\;i\neq 6$$

(8) $$\begin{array}{c} x_{2}=\{\left(R_{11}^{\left(0-6\right)},R_{12}^{\left(0-6\right)},\cdots ,R_{32}^{\left(0-6\right)},R_{33}^{\left(0-6\right)}\right),\cdots ,\left(R_{11}^{\left(i-6\right)},R_{12}^{\left(i-6\right)},\cdots ,R_{32}^{\left(i-6\right)},R_{33}^{\left(i-6\right)}\right),\cdots ,\\ \left(R_{11}^{\left(10-6\right)},R_{12}^{\left(10-6\right)},\cdots ,R_{32}^{\left(10-6\right)},R_{33}^{\left(10-6\right)}\right){\}}^{T}\;\forall i=0,1,\cdots ,10\;\mathrm{and}\;i\neq 6 \end{array}$$

Given the training set $\left\{x_{2}^{\left(i\right)},i=1,2,\cdots ,m\right\}$ and the number of classes $K_{2}$, the FCM aimed to obtain $K_{2}$ clusters and corresponding cluster center to make the dissimilarity of the samples divided into the same cluster become the smallest. The cost function is defined as $J_{f}=\sum_{j=1}^{K_{2}}\sum_{i=1}^{m}{\left[\mu_{j}\left(x_{2}^{\left(i\right)}\right)\right]}^{b}\left|\right|x_{2}^{\left(i\right)}-m_{j}{\left|\right|}^{2}$, where $m_{j}$ is the cluster center of the jth cluster, and $\mu_{j}\left(x_{2}^{\left(i\right)}\right)$ is the membership degree that the ith sample belongs to the jth cluster and $\sum_{j=1}^{K_{2}}\mu_{j}\left(x_{2}^{\left(i\right)}\right)=1$. *b* is the weight index, which controls the importance of the membership degree, and mostly *b* is set to 2.

Let the derivative of $J_{f}$ with respect to $m_{j}$ and $\mu_{j}\left(x_{2}^{\left(i\right)}\right)$ equal 0 respectively, and we can deduce the following

(9) $$m_{j}=\frac{\sum_{i=1}^{m}{\left[\mu_{j}\left(x_{2}^{\left(i\right)}\right)\right]}^{b}x_{2}^{\left(i\right)}}{\sum_{i=1}^{m}{\left[\mu_{j}\left(x_{2}^{\left(i\right)}\right)\right]}^{b}},\forall j=1,2,\cdots ,K_{2}$$

(10) $$\mu_{j}\left(x_{2}^{\left(i\right)}\right)=\frac{\left|\right|x_{2}^{\left(i\right)}-m_{j}{\left|\right|}^{\frac{-2}{b-1}}}{\sum_{s=1}^{K_{2}}\left|\right|x_{2}^{\left(i\right)}-m_{j}{\left|\right|}^{\frac{-2}{b-1}}}\;\;\forall i=1,2,\cdots ,m,j=1,2,\cdots ,K_{2}$$

By repeating iteration up to that $J_{f}$ is converged, the optimal classification $\left\{\mu_{j}\left(x_{2}^{\left(i\right)}\right),i=1,2,\cdots ,m,j=1,2,\cdots ,K_{2}\right\}$ and corresponding cluster centers $\left\{m_{j},j=1,2,\cdots ,K_{2}\right\}$ are obtained.

### 3.2. Posture Recognition

In the recognition stage, as shown in Algorithm 1, nine components of the rotation matrix in the local waist IMU coordinate system $x={\left\{R_{11}^{\left(6\right)},R_{12}^{\left(6\right)},R_{13}^{\left(6\right)},R_{21}^{\left(6\right)},R_{22}^{\left(6\right)},R_{23}^{\left(6\right)},R_{31}^{\left(6\right)},R_{32}^{\left(6\right)},R_{33}^{\left(6\right)}\right\}}^{T}$ were input into the BP-ANN classifier and the category with the max probability and larger than the given threshold $t_{1}$ was regarded as the output result. Then 90 components of the rotation matrix in the rest local IMU coordinate systems relative to the waist IMU coordinate system $x_{2}={\left\{\left(R_{11}^{\left(0-6\right)},R_{12}^{\left(0-6\right)},\cdots ,R_{32}^{\left(0-6\right)},R_{33}^{\left(0-6\right)}\right),\cdots ,\left(R_{11}^{\left(10-6\right)},R_{12}^{\left(10-6\right)},\cdots ,R_{32}^{\left(10-6\right)},R_{33}^{\left(10-6\right)}\right)\right\}}^{T}$, were input into the corresponding FCM classifier and the posture with max membership degree and larger than the given threshold $t_{2}$ were regarded as the recognition result of the current data frame.

**Algorithm 1: **Pseudocode of recognition method

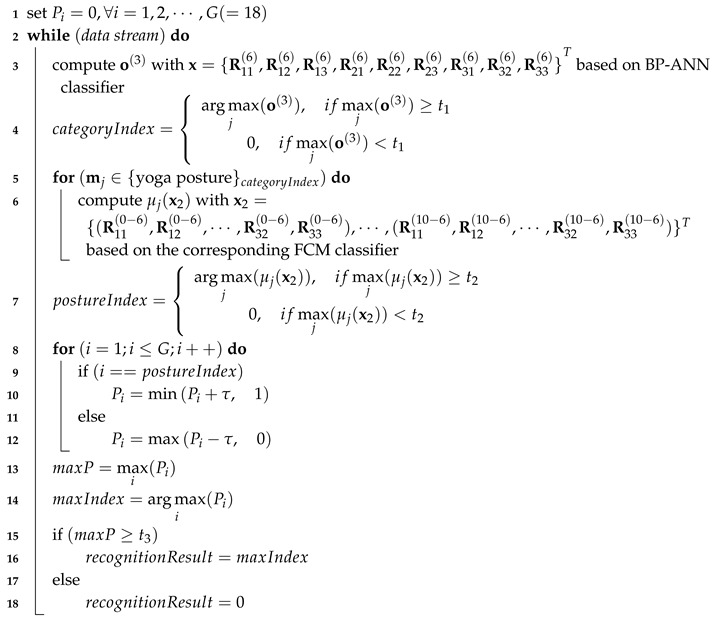



Since yoga postures are recognized in every sample period, some isolated frames may be recognized as posture or noisy result within a posture instance. In order to eliminate these isolated recognized results, the final recognition result was determined with cumulative probability. Before recognition of a posture instance, the likelihood of all postures was set to zero in advance. When the result of the current data frame was recognized, the likelihood of recognition result added an updating value τ, while the likelihood of other postures minused the same updating value. In this paper, τ was set to 0.1. When the likelihood of a posture was greater than the given threshold $t_{3}$, the posture was output as the final recognition result. Such a method could significantly eliminate isolated frame and noisy result, and further improve the system robustness.

## 4. Posture Evaluation

**Figure 4:**
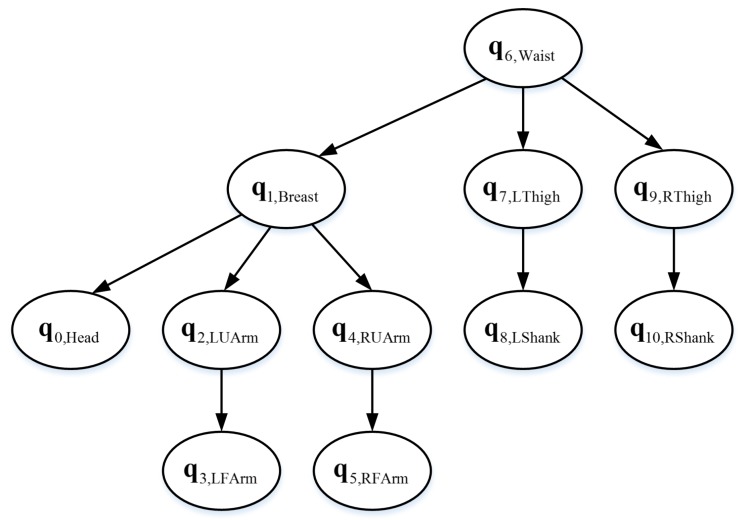
The schematic of constructed Bayesian network for evaluation. The waist part is the parent point of other body parts, and upper body and lower body are built based on the connection of the rest parts from the waist.

Since human posture was performed by multiple joints, it was natural to focus on joints to provide feedback and guidance, such as “straighten your left forearm” to guide elbow joint, and “lift up your left hand” to guide shoulder joint. The joints were formed by each body parts with the corresponding connected body parts, and therefore, it is reasonable to regard the relative posture of each body part respect to the connected body part as the criterion for evaluating the joints or the corresponding body part. For example, the relative posture of forearm relative to upper arm could be used to evaluate the standard degree of forearm. According to the advice of professional yoga instructors, most yoga postures require the waist to be stable, and at the same time, the lower body movement is performed by the lower limb joint chain of hip-knee-ankle and the upper body movement is performed by the upper limb joint chain of thoracic–shoulder–elbow–wrist or thoracic–neck. For example, grasshoppers (shown in the eleventh subfigure in Figure 2) needs the waist to be stable and requires the head, arm, and leg to be lifted up as much as possible. Based on this theory, a Bayesian network is constructed to evaluate the standard degree of various parts of the body, as shown in Figure 4. The standard degree of the body part is quantitatively evaluated by the conditional probability of Gaussian variable corresponding to each body part relative to Gaussian variable corresponding to the connected body part. Each body part is a four-dimensional Gaussian model based on the quaternion data of the fixed IMU coordinate system relative to the waist IMU coordinate system.

Take the LUArm as an instance. Given the training set $\left\{s^{\left(i\right)},i=1,2,\cdots ,m,s\in R^{4}\right\}$, where s is the quaternion of the local LUArm IMU coordinate system relative to the local waist IMU coordinate system, and $\left\{t^{\left(i\right)},i=1,2,\cdots ,m,t\in R^{4}\right\}$, where t is the quaternion of the local breast IMU coordinate system relative to the local waist IMU coordinate system, the mean vector $\mu_{s,t}$ and covariance matrix $\sum_{s,t}$ of combined Gaussian model of LUArm and breast could be calculated as follows

(11) $$\mu_{s,t}=\frac{\sum_{i=1}^{m}z^{\left(i\right)}}{m}=\left(\begin{array}{c} \mu_{s}\\ \mu_{t} \end{array}\right)$$

(12) $$\sum_{s,t}\left(p,q\right)=\frac{\sum_{i=1}^{m}\left(z^{\left(i\right)}\left(p\right)-\mu_{s,t}\left(p\right)\right)\left(z^{\left(i\right)}\left(q\right)-\mu_{s,t}\left(q\right)\right)}{m}\;\;\forall p,q=1,2,\cdots ,8$$

where $z^{\left(i\right)}={\left(s^{\left(i\right)},t^{\left(i\right)}\right)}^{T}\forall i=1,2,\cdots ,m$, $\mu_{x}$ and $\mu_{y}$ are the mean vector of LUArm and breast Gaussian model, respectively.

Moreover, the density function of the combined Gaussian model p(s,t) could be expressed as

(13) $$\begin{array}{c} \ln p\left(s,t\right)=\ln\left\{\frac{1}{\sqrt{{\left(2\pi \right)}^{8}|\sum_{s,t}|}}\exp(-\frac{1}{2}{\left[\left(\begin{array}{c} s\\ t \end{array}\right)-\mu_{s,t}\right]}^{T}\sum_{s,t}^{-1}\left[\left(\begin{array}{c} s\\ t \end{array}\right)-\mu_{s,t}\right]\right)\}\\ =-\frac{1}{2}t^{T}B_{22}t+A_{2}^{T}t-\frac{1}{2}s^{T}B_{11}s+A_{1}^{T}s+C \end{array}$$

where $\sum_{s,t}^{-1}=\left(\begin{array}{cc} B_{11} & B_{12}\\ B_{21} & B_{22} \end{array}\right)$, and $B_{11}$ and $B_{22}$ are the inverse covariance matrix of LUArm and Breast Gaussian model, $A_{1}=B_{11}\mu_{s}+B_{12}\mu_{t}$, $A_{2}=B_{22}\mu_{t}-B_{21}\left(s-\mu_{s}\right)$, *C* is a constant.

The edge density function of breast Gaussian model p(t) can also be deduced as $\ln p\left(t\right)=-\frac{1}{2}t^{T}B_{22}t+\mu_{t}^{T}B_{22}t+C_{1}$, where $C_{1}$ is a constant.

As a result, the density function of LUArm Gaussian model relative to breast Gaussian model p(s|t) is

(14) $$\ln p\left(s|t\right)=\ln p\left(s,t\right)-\ln p\left(t\right)=-\frac{1}{2}s^{T}B_{11}s+A_{3}^{T}s+C_{2}\left(t\right)$$

where $A_{3}=B_{11}\mu_{s}-B_{12}\left(t-\mu_{t}\right)$, and $C_{2}\left(t\right)$ is independent of s. Hence, the conditional probability model of LUArm Gaussian model relative to breast Gaussian model is $\left(s|t\right)∼N\left(\mu_{s|t},\sum_{s|t}\right)$, where $\mu_{s|t}=\mu_{s}-B_{11}^{-1}B_{12}\left(t-\mu_{t}\right)$, $\sum_{s|t}=B_{11}^{-1}$. The conditional probability of LUArm relative to breast could be calculated by $N(\mu_{s|t},\sum_{s|t})$, and $P(||S-\mu_{s|t}||\leq ||s-\mu_{s|t}||)$ will be the criterion to quantitatively evaluate the standard degree of the LUArm part.

If the conditional probability of Gaussian variable corresponding to LUArm relative to Gaussian variable corresponding to breast was smaller than a given threshold, it was regarded as a nonstandard part, and then the specific guidance was provided to the yoga learner, including the correction orientation and extent. The actual quaternion of LUArm relative to the conditional probability model of LUArm relative to breast $q_{2|1,\mathrm{actual}-\mathrm{model}}$ was calculated.

(15) $$q_{2|1,\mathrm{actual}-\mathrm{model}}=\mu_{s|t}^{-1}⊗q_{2-6}⟹\left(\begin{array}{ccc} R_{11}^{\left(2|1\right)} & R_{12}^{\left(2|1\right)} & R_{13}^{\left(2|1\right)}\\ R_{21}^{\left(2|1\right)} & R_{22}^{\left(2|1\right)} & R_{23}^{\left(2|1\right)}\\ R_{31}^{\left(2|1\right)} & R_{32}^{\left(2|1\right)} & R_{33}^{\left(2|1\right)} \end{array}\right)$$

Consequently, the differences between actual posture and standard model can be acquired. In order to provide easily-accepted guidance to the yoga learner, the differences are transformed to the guidance information in the absolute coordinate of skeleton animation according to the posture category. As for LUArm and RUArm, the guidance orientation includes forward, backward, upward, and downward, which are determined by the corresponding body part relative to breast. As for head, LThigh, and RThigh, the guidance orientation includes forward, backward, leftward, and rightward, which are determined by the corresponding body part relative to waist. As for LFArm, RFArm, LShank, and RShank, the guidance orientation included bend and stretch, which were determined by the corresponding body part relative to the connected body part respectively. Additionally, the guidance extent was defined as the angle between the actual orientation and standard model orientation.

If the posture belonged to the ”stand category”, the angle difference of LUArm in the fore-and-back orientation and up-and-down orientation, $d_{\mathrm{fore}-\mathrm{and}-\mathrm{back}}$ and $d_{\mathrm{up}-\mathrm{and}-\mathrm{down}}$, could be calculated by

(16) $$d_{\mathrm{fore}-\mathrm{and}-\mathrm{back}}=\mathrm{sign}\left(-R_{21}^{\left(2|1\right)}\right)\arccos\left(\frac{R_{11}^{\left(2|1\right)}}{\sqrt{{\left(R_{11}^{\left(2|1\right)}\right)}^{2}+{\left(R_{21}^{\left(2|1\right)}\right)}^{2}}}\right)$$

(17) $$d_{\mathrm{up}-\mathrm{and}-\mathrm{down}}=\mathrm{sign}\left(-R_{31}^{\left(2|1\right)}\right)\arccos\left(\frac{R_{11}^{\left(2|1\right)}}{\sqrt{{\left(R_{11}^{\left(2|1\right)}\right)}^{2}+{\left(R_{31}^{\left(2|1\right)}\right)}^{2}}}\right)$$

if $d_{\mathrm{fore}-\mathrm{and}-\mathrm{back}}\geq 0$, the guidance put LUArm backward until the difference was within an acceptable range, and vice versa. Similarly if $d_{\mathrm{up}-\mathrm{and}-\mathrm{down}}\geq 0$, the guidance put LUArm downward, and vice versa. Likewise, the guidance about the RUArm, head, LThigh, and RThigh could be calculated by the above method.

If the nonstandard body parts included LFArm, the joint angle was the guidance criterion, and the difference in the bend-and-stretch orientation $d_{\mathrm{bend}-\mathrm{and}-\mathrm{stretch}}$ could be calculated by

(18) $$d_{\mathrm{bend}-\mathrm{and}-\mathrm{stretch}}=\arccos\left({\left(V_{\mathrm{model},3-2}\right)}_{x}\right)-\arccos\left({\left(V_{\mathrm{actual},3-2}\right)}_{x}\right)$$

where Vmodel,3−2=Im{(q2−6−1⊗μ3|2)⊗(0,1,0,0)⊗(q2−6−1⊗μ3|2)−1} and Vactual,3−2=Im{(q2−6−1⊗q3−6)⊗(0,1,0,0)⊗(q2−6−1⊗q3−6)−1}.

If $d_{\mathrm{bend}-\mathrm{and}-\mathrm{stretch}}\geq 0$, the guidance bent the left elbow joint angle, and vice versa. Similarly, the guidance about the RFArm, LShank, and RShank could also be calculated.

Similarly, if the posture belonged to another category, the guidance calculated by the above method was transformed into the orientation from the trainer’s perspective.

## 5. Results and Discussion

### 5.1. Posture Recognition Results

In this section, 30% data were picked randomly from all subjects to train the ANN classifier and FCM classifier, and the remaining 70% data were used for testing. In the training stage, three posture instances were picked randomly from each kind of postures of all subjects. Each data frame was input into the ANN classifier to obtain the classified category, and then was input into the corresponding FCM classifier to obtain the recognition result. The test result for each posture with data frame was shown in Table 1. The accuracy of postures recognition was between 70% and 100%, and the average accuracy was 89.34%. In order to eliminate these isolated recognized results and further improve the accuracy, the proposed recognition method was adopted with cumulative probability according to Algorithm 1. The test result in the posture instance recognition was shown in Table 2. The average recognition accuracy was 95.39%, which was highly improved. As for some postures, such as posture 11, the recognition accuracy was increased a lot. It is easy to see that posture 11 needed the waist to be stable and required the head, arm, and leg to be lifted up as much as possible. In fact, some trainers couldn’t maintain this posture for an instance time (6–10 s) and it sometimes happened that the trainer temporarily put down their hands and legs for rest. The new posture that the trainer puts down their hands and legs was unlike the rest of the yoga postures, and therefore it easily caused the recognition probabilities of 18 yoga postures were smaller than the given threshold, and finally the current data frame was recognized as noisy result within a posture instance. However, our recognition methods (Algorithm 1) adopted cumulative probability to eliminate these isolated recognized noisy results, and thus the recognition accuracy of posture 11 was greatly improved. As for some postures, such as posture three, the recognition accuracy was decreased a little. Posture three required the trainer to stand on one leg. Similarly, it was hard for some trainers to keep balance in posture three, and it also sometimes happened that the trainer lost balance and had to temporarily put down their legs. The difference was that the new posture in which the trainer put down their legs was like posture one, and therefore the current data frame was easily recognized as posture one. In our recognition methods, the final recognition result was determined with cumulative probability. Hence, some instances of posture three were recognized as posture one, which caused the recognition accuracy to decrease a little.

### 5.2. Posture Evaluation Results

In this section, 30% data were picked randomly as the training database from subject three, subject four, and subject six, who performed yoga posture better relatively. The standard and nonstandard testing database were the yoga posture data performed standardly and deliberately non-standardly by another new trainer respectively. The nonstandard testing database includes 12 nonstandard postures, and each posture was corresponding to one or two nonstandard body parts.

**Figure 5:**
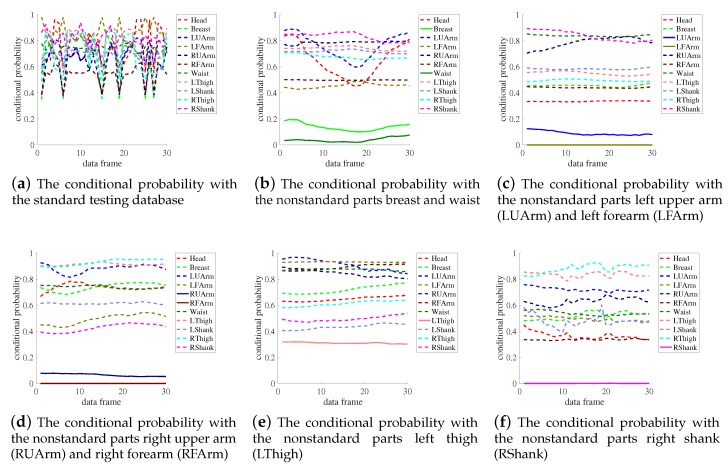
The conditional probability of 11 body parts of stand deep breathing computed by the trained Bayesian network with the first 30 frames in the testing database. Subfigure (**a**) is the standard posture evaluation results for comparison. Subfigure (**b**–**f**) are the nonstandard posture evaluation results, where the conditional probabilities of the nonstandard parts are marked with a solid line. The nonstandard parts involve breast, waist, forearm, upper arm, thigh, and shank.

In the training stage, the quaternion data of 11 IMUs in the training set were used for training the Bayesian network. In the evaluation stage, both standard and nonstandard postures were used for testing. Take stand deep breathing as an instance. As shown in Figure 5, the subfigure (a) showed the conditional probability of 11 body parts with the standard testing database, and we can see that probability of 11 body parts were almost all greater than 0.5. The subfigure (b–f) were the nonstandard posture evaluation results, where the conditional probabilities of the nonstandard parts were marked with solid line. The nonstandard parts involved breast, waist, forearm, upper arm, thigh, and shank. It’s easy to see that the probabilities of nonstandard parts (marked with solid line) were almost all smaller than 0.3, while the probabilities of standard parts (marked with dotted line) were almost all greater than 0.5. Thus one can see that the nonstandard parts and standard parts could be effectively separated.

**Figure 6:**
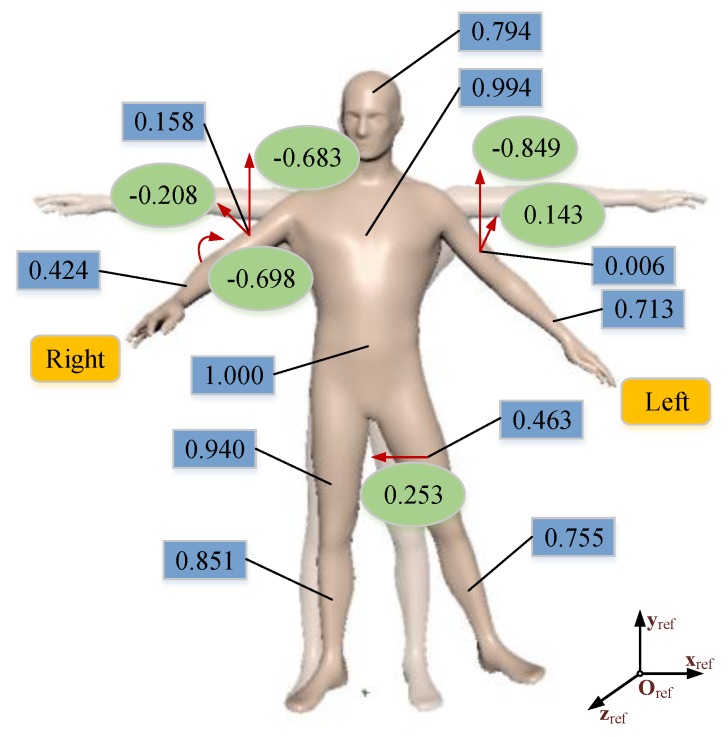
Illustration of yoga posture evaluation and guidance based on the proposed evaluation method. The numbers in the blue rectangular box are evaluation probabilities of 11 body parts of the actual posture (the materialized figure) relative to the standard posture (the fictitious figure). The numbers in the green ellipse box and the red arrows are angular differences (rad) and guidance orientations, respectively.

The posture guidance results are shown in Figure 6. One can see that the LUArm and RUArm of the actual posture (the materialized figure) were lower than the standard posture (the fictitious figure). The RFArm was not straight, and the LThigh was deviated to left from the standard posture. The numbers in the blue rectangular box are evaluation probabilities of 11 body parts, and the probabilities of the above nonstandard parts are fairly low. Accordingly, the evaluation results are consistent with the actual nonstandard body parts. The numbers in the green ellipse box and red arrows are angular differences (rad) and guidance orientations calculated by the proposed method respectively. The guidance results were also reasonable, and as stated before, the guidance were provided to the learner with an easily-accepted language, such as “lift up your left arm”, “straighten your right forearm”, and “put your left leg to right”. Hence, the Bayesian network can be an effective evaluation method to determine the nonstandard parts in a human body posture.

**Figure 7:**
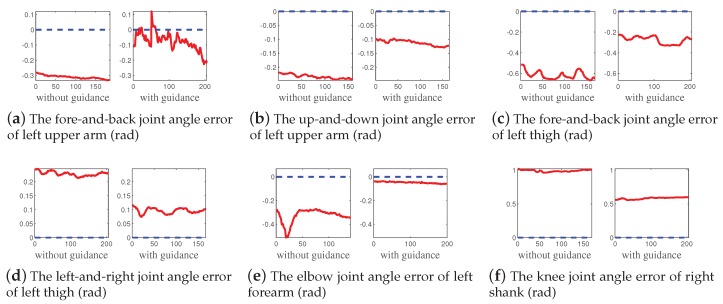
The yoga posture performance comparison of posture five between without guidance and with guidance. The errors between the various joint angles and standard model were calculated and marked as red line. The blue line was the reference line for intuitively comparison, which means the error equals 0. The joint angles involve the fore-and-back and up-and-down joint angle of left upper arm, the fore-and-back and left-and-right joint angle of left thigh, the elbow joint angle of left forearm, and the knee joint angle of right shank.

In order to evaluate our proposed method, the comparative test was conducted. We tested separately the trainers’ yoga posture performance in the condition of without and with guidance provided by our proposed method. Figure 7 showed the yoga posture performance comparison of posture five between without guidance and with guidance. The errors between the various joint angles and standard model were calculated and marked as red line. The blue line was the reference line for intuitively comparison, which means the error equals 0. We can see that, with guidance, the joint angle errors significantly decreased. For example, the fore-and-back and up-and-down joint angle errors of left upper arm declined about 0.2 rad and 0.1 rad, respectively. As for especially non-standard body parts, such as the knee joint angle error of right shank, the joint angle error could even decline by about 0.5 rad. Therefore, the proposed method was effective to provide feedback to guide the trainer to perform properly.

### 5.3. Posture Recognition Robustness Evaluation

When we were doing data analysis, we found that the body shapes of different trainers performing the same yoga posture were quite different. For quantifying this difference, we evaluated the angle between breast and LThigh, and the angle between waist and LThigh, which could typically reflect the training core of left semilunar. The results are shown in Table 3. The evaluated angles were quite different among subjects and instances of a specific subject. The range of the angles among subjects could reach almost 0.857 rad and 1.077 rad respectively. Additionally, the range among instances of the same subject could maximally reach almost 0.404 rad and 1.224 rad, respectively. These large differences could cause large intra-class distance and small interclass distance. Therefore, it was difficult to linearly distinguish yoga postures in the original feature space, and accordingly, nonlinear mapping was adopted. As mentioned before, we proposed a two-stage classifier of BP-ANN and FCM to deal with the above difficulties. The recognition result shows that the two-stage classifier could distinguish 18 yoga postures effectively and achieve good accuracy. By comparing the recognition results of Table 1 and Table 2, one can see that the proposed recognition method could effectively eliminate isolated recognized results and noisy results with cumulative probability, and further improve the recognition accuracy.

### 5.4. The Comparison of Posture Membership and Evaluation Probability

As shown in Table 4, the comparison of the posture membership and evaluation probability was conducted. The second column shows the mean membership of the left semilunar of eight subjects based on the FCM classifier trained by the 30% data randomly picked from subject three, subject four, and subject six. The third column shows the mean evaluation probability of LThigh relative to waist of eight subjects calculated by the Bayesian network trained by the same database. Subject two and subject five performed more standardly according to the evaluation results by Bayesian network (marked with *), and meanwhile, their memberships computed by the FCM classifier were also very high. Both criteria represent the standard degree of left semilunar in some sense. Hence, one can see that the evaluation results are in line with the membership results, and therefore the evaluation results are reliable and accurate.

### 5.5. Comparison between the Proposed Method and Other Methods in the Literature

Table 5 shows a comparison among a few methods in the literature and our proposed method. Camera-based methods, such as deep learning [26] and star skeleton [27], could achieve a high accuracy in posture recognition and the subjects could perform yoga posture in a more comfort and natural way, but they only modeled and recognized yoga postures without body folding. Actually, most yoga postures had body folding, such as postures 10, 13, and 18 shown in Figure 2, which could produce image occlusion, and they were hard to recognize via cameras. Moreover, though our accuracy was lower than [26,27], it should be noticed that we have modeled more postures and tested more instances. Additionally, they haven’t applied the yoga identification system to evaluating and guiding the yoga postures. YogaST [22], adaboost algorithm [28] and OpenPose [29] were also widely used in yoga posture recognition, but they similarly only modeled a few yoga posture without body folding and the recognition accuracy was lower than our proposed method. In addition, they also haven’t applied the yoga recognition system to evaluating and guiding the yoga postures. Template star skeleton [30] was used to recognize 12 yoga postures via two cameras, and visual feedback was adopted for providing guidance to subjects. Although our wearable device was less comfortable than [30], we have modeled more yoga postures including body folding and achieved a higher accuracy. Besides, the posture evaluation method via IMUs was more precise than that via cameras, since it may be hard to acquire precise joint angles via the key points and contour of yoga postures extracted from images. Hence, image sensors may not be suitable for precise yoga posture evaluation and guidance, and therefore we chose more precise IMUs to model yoga postures. Moreover, with the miniaturization and integration of IMU, the wearable experience of IMUs will be further improved. Motion replication [31] was another yoga posture recognition and evaluation method by using IMUs, but they adopted more IMUs and haven’t applied the yoga posture recognition and evaluation system to actual testing.

## 6. Conclusions

In this paper, we proposed a full-body posture modeling and quantitative evaluation method to recognize and evaluate yoga postures and provide guidance to learner. BP-ANN and FCM were employed to construct a two-stage classifier to model and recognize full-body postures. BP-ANN was adopted as the first classifier to divide yoga postures into different categories due to its powerful nonlinear processing ability, and FCM was adopted as the second classifier to classify the postures in a category for the flexible fuzzy partition. The two-stage classifier could deal with the posture differences among subjects effectively and improve recognition results with low computing cost. In addition, we also proposed a recognition method to eliminate isolated recognized results and noisy results with cumulative probability, and further improve the recognition accuracy.

The quaternion data measured by IMU fixed on each body part was regarded as a multidimensional Gaussian variable to build a Bayesian network. The conditional probability of the Gaussian variable corresponding to each body part relative to the Gaussian variable corresponding to the connected body part was used to quantitatively evaluate the standard degree of the body part. Furthermore, guidance was provided to learner with an easily-accepted language, including correcting orientation and extent.

To evaluate the proposed methods, the posture database with totally 211,643 data frames and 1831 posture instances, including 18 common yoga postures, was collected from 11 subjects. Both the data frame recognition and the posture instance recognition tests were conducted. In the data frame recognition test, 30% data were picked randomly from the database to train BP-ANN and FCM classifiers, and the recognition accuracy of the remaining 70% data was 89.34%. In the posture instance recognition test, the recognition accuracy of the same test database was 95.39% by employing the recognition method.

As for posture evaluation, 30% data were picked randomly from subject three, subject four, and subject six, to train Bayesian network, and both the standard posture and nonstandard posture evaluation tests were conducted. The probabilities of nonstandard parts were almost all smaller than 0.3, while the probabilities of standard parts were almost all greater than 0.5. One can see that the nonstandard parts and standard parts could be effectively separated. Moreover, the posture guidance results show that the provided corrections were also reasonable and effective. We also tested separately the trainers’ yoga posture performance without and with guidance provided by our proposed method. The results showed that, with guidance, the joint angle errors significantly decreased. Hence, the Bayesian network could be an effective and reasonable evaluation method to find out the nonstandard parts of a human body posture, and further provide feedback to guide the trainer to perform properly. We believe that our proposed method could be applied to any full-body postures which need evaluation and feedback, including exercises like tai chi, full-body movements like human daily behaviors, and human postures like yoga.

## Figures and Tables

**Table 1:** Posture recognition result for each posture with data frame in the test database.

Posture Index	Correctly Recognized Data Frames	Total Frames	Accuracy (%)
1	9351	9384	99.65
2	8729	9192	94.96
3	8805	9459	93.09
4	6278	8746	71.78
5	8792	8792	100.00
6	7414	9131	81.20
7	8654	8663	99.90
8	9386	9499	98.81
9	8630	8630	100.00
10	6937	8596	80.70
11	6171	8252	74.78
12	8183	8772	93.29
13	6260	8581	72.95
14	6043	8563	70.57
15	9202	9202	100.00
16	9021	9021	100.00
17	8788	8788	100.00
18	6097	8505	71.69
Total	142,741	159,776	89.34

**Table 2:** Posture recognition result for each posture with instance in the test database.

Posture Index	Correctly Recognized Posture Instances	Total Instances	Accuracy (%)
1	76	77	98.70
2	72	77	93.51
3	67	77	87.01
4	56	63	88.89
5	77	77	100.00
6	60	77	77.92
7	77	77	100.00
8	75	77	97.40
9	77	77	100.00
10	63	63	100.00
11	55	59	93.22
12	71	77	92.21
13	54	60	90.00
14	55	56	98.21
15	77	77	100.00
16	77	77	100.00
17	77	77	100.00
18	56	56	100.00
Total	1222	1281	95.39

**Table 3:** The angles differences comparison between breast and LThigh, and between waist and LThigh of left semilunar performed by 11 subjects.

Subjects	Angles between Breast and LThigh (rad)			Angles between Waist and LThigh (rad)		
	Mean	Standard Deviation	Range	Mean	Standard Deviation	Range
1	0.460	0.043	0.202	2.091	0.074	0.326
2	0.585	0.059	0.259	2.119	0.049	0.286
3	0.509	0.070	0.262	2.396	0.053	0.184
4	0.568	0.039	0.190	1.705	0.482	1.224
5	0.271	0.054	0.225	2.025	0.065	0.268
6	0.089	0.039	0.190	1.660	0.107	0.431
7	0.730	0.064	0.222	2.306	0.101	0.332
8	0.516	0.033	0.137	2.068	0.038	0.157
9	0.594	0.078	0.404	1.990	0.120	0.524
10	0.394	0.063	0.268	2.092	0.115	0.419
11	0.946	0.109	0.365	2.737	0.110	0.389
Range of all subjects	0.857	-	-	1.077	-	-

**Table 4:** The membership of the left semilunar and the evaluation probability of LThigh relative to waist in the left semilunar.

Subjects	Mean Membership Value	Mean Evaluation Probability of LThigh Relative to Waist
1	0.195	0.369
2 *	0.465 *	0.818 *
5 *	0.532 *	0.700 *
7	0.199	0.472
8	0.197	0.414
9	0.202	0.434
10	0.190	0.382
11	0.172	0.339

* The subjects who performed yoga postures more standardly than others.

**Table 5:** Comparison of several different yoga recognition and evaluation methods.

Method	Sensors	Wearable Experience	Yoga Posture Number	Posture Type	Posture Instances/Frames	Posture Recognition Accuracy	Posture Evaluation and Guidance	Posture Evaluation Precision
Deep learning [26]	a RGB webcam	comfort	6	without body folding	929 instances	98.92%	-	-
Star skeleton [27]	a Kinect	comfort	12	without body folding	300 instances	99.33%	-	-
YogaST [22]	two Kinects	comfort	3	without body folding	27,735 frames	82.84%	-	-
Adaboost algorithm [28]	a depth sensor- based camera	comfort	6	without body folding	5685 frames	94.78%	-	-
OpenPose [29]	a RGB camera	comfort	6	without body folding	-	-	-	-
Template star skeleton [30]	two cameras	comfort	12	without body folding	29,260 frames	94.30%	visual feedback	less precise
Motion replication [31]	16 IMUs and 6 tactors	less comfort	-	-	-	-	visual and haptic feedback	precise
The proposed method	11 IMUs	less comfort	18	including body folding	1281 instances /159,776 frames	95.39%	voice feedback	precise
